# Dual blocking of PI3K and mTOR signaling by DHW‐221, a novel benzimidazole derivative, exerts antitumor activity in human non‐small cell lung cancer

**DOI:** 10.1002/ctm2.514

**Published:** 2021-09-26

**Authors:** Xiaochun Qin, Mingyue Liu, Yuting Wu, Shu Wang, Siheng Lian, Hui Jia, Qiong Wu, Huaiwei Ding, Qingchun Zhao

**Affiliations:** ^1^ Department of Life Science and Biochemistry Shenyang Pharmaceutical University Shenyang China; ^2^ Department of Pharmacy General Hospital of Northern Theater Command Shenyang China; ^3^ Key Laboratory of Structure‐Based Drug Design and Discovery of Ministry of Education Shenyang Pharmaceutical University Shenyang China

Dear Editor,

We previously designed and synthesized a novel benzimidazole compound, DHW‐221.^1^ Here, we evaluated its anti‐NSCLC activity as a PI3K/mTOR dual‐target inhibitor and comprehensively described for the first time the mechanism underlying the PI3K/AKT/mTOR signaling pathway‐mediated antitumor effects of DHW‐221. Our findings indicated that the antitumor activity of DHW‐221 was significantly stronger than that of NVP‐BEZ235, a dual PI3K/mTOR inhibitor currently undergoing a phase II clinical trial.

The PI3K/AKT/mTOR pathway is a key mediator of receptor tyrosine kinase signal transduction. The PI3K family can be divided into three categories.^2^ Class I PI3K and mTOR, sharing structural homology in the kinase domain,^3^ are considered important targets for malignancy therapy.^4^ Thus, the thought of blocking the PI3K/AKT/mTOR pathway by double inhibition of PI3K/mTOR was proposed.^5^ Herein, we systematically elucidated the anti‐NSCLC effects associated with DHW‐221‐mediated dual PI3K/mTOR inhibition and identified the mechanism underlying this effect both in vitro and in vivo.

First, we identified potential targets of DHW‐221. Molecular docking indicated that DHW‐221 fitted well into the PI3K‐ATP binding pocket and formed hydrogen bonds with Gln859 and Arg770. For mTOR, DHW‐221 precisely overlapped with it and formed two more hydrogen bonds (Val2240 and Thr2245) than NVP‐BEZ235 (Figure 1A). Docking results with PI3Kβ/γ/δ are shown in Figure S1. Subsequent kinase assay suggested that DHW‐221 significantly inhibits the kinase activity of the main PI3K isoforms^1^ (IC_50_: PI3Kα,0.5 nM; PI3Kβ,1.9 nM; PI3Kγ,1.8 nM; PI3Kδ,0.74 nM), as well as that of mTOR (IC_50_: 3.9 nM) (Table S1). Cellular thermal shift assay was used to further evaluate the binding mode, the thermal stability of DHW‐221 combined with PI3K/mTOR was concentration‐dependently enhanced (Figure 1B). Additionally, we knocked down PI3K in HCC827 cells (Figure 1C) and compared how DHW‐221, NVP‐BEZ235, and wortmannin (a PI3K inhibitor) treatments affected AKT and mTOR protein phosphorylation levels. No significant difference in p‐AKT levels was found with any of the treatments (Figure 1D), suggesting that DHW‐221 likely targeted PI3K. Conversely, compared with wortmannin, p‐mTOR expression was significantly decreased after DHW‐221 and NVP‐BEZ235 intervention, implying that DHW‐221 may target mTOR. Finally, western blot and immunofluorescence analysis confirmed the effect of DHW‐221 on the expression of PI3K/AKT/mTOR pathway‐related proteins both in vitro and in vivo (Figure 1E–G). Combined, these results suggested that DHW‐221 inhibited PI3K/AKT/mTOR pathway activation through targeting PI3K/mTOR. The effects of DHW‐221 on notch pathway are provided in Result S1.

**FIGURE 1:**
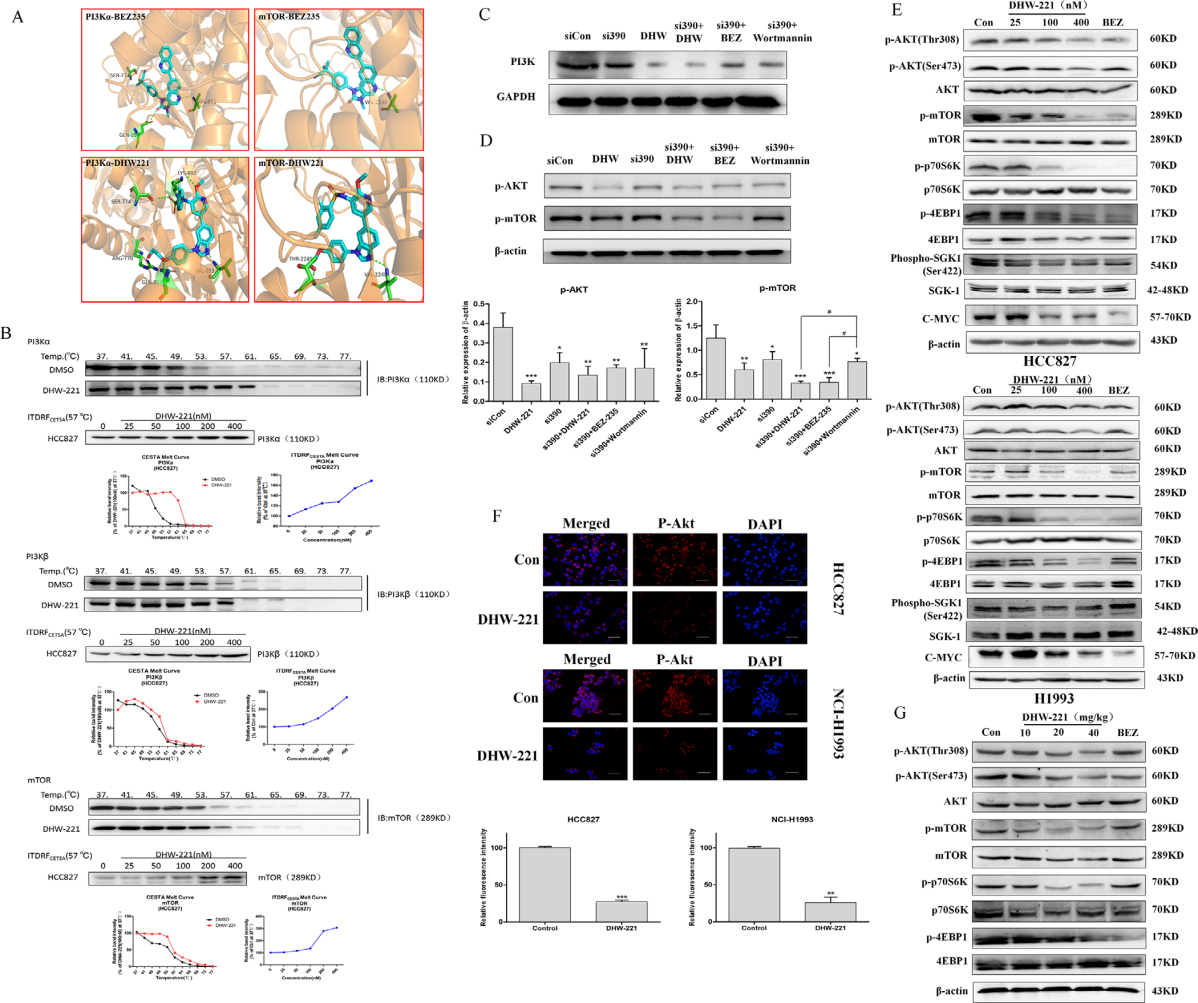
DHW‐221 is a PI3K/mTOR dual‐target inhibitor and inhibits the PI3K/AKT/mTOR signaling pathway. (A) Predicted binding modes for DHW‐221 (blue stick) with PI3Kα (PDB code: 4JPS) and mTOR (PDB code: 4JT6). Hydrogen bonds are shown as green dashed lines. Key residues for DHW‐221 and BEZ235 interaction are highlighted. (B) A cellular thermal shift assay (CETSA) and an isothermal dose–response fingerprinting (ITDRF)cetsa were used to evaluate the thermal stability of DHW‐221 bound to PI3Kα/β or mTOR. (C, D) The expression of PI3K, p‐AKT, and p‐mTOR in transfected NSCLC cells was assessed by western blot. (E, G) Western blot was also used to evaluate the effects of DHW‐221 on PI3K/AKT/mTOR pathway‐related proteins in non‐small cell lung cancer (NSCLC) cells and tissues (*n* = 6). (F) Immunofluorescence images of p‐AKT protein expression following DHW‐221 treatment. Anti‐rabbit p‐AKT (Ser473) antibody was used for labeling and the nuclei were stained with DAPI; scale bar, 50 µm. Mean ± SD; *p < 0.05, **p < 0.01, ***p < 0.001 versus the control (model). One‐way analysis of variance followed by Tukey's post hoc multiple‐comparisons test

To investigate the effect of DHW‐221 in vitro, SRB assay was performed. DHW‐221 exhibited excellent inhibitory activity concentration‐ and time‐dependently (Figure 2B) without affecting the normal cells (Figure S3), and IC_50_ is shown in Table S2. Additionally, the long‐term efficacy of DHW‐221 was evaluated and confirmed a better antiproliferation role than NVP‐BEZ235 (Figure 2C–E). Figure 2F investigates the concentration‐dependently inhibition of DHW‐221 on protein synthesis in cells. HCC827 xenograft models in both subcutaneous and orthotopic were used to further evaluate its antitumor potency in vivo. As illuminated in Figure 2G–P, DHW‐221 apparently inhibited tumor growth without affecting body weight (Figure 2J, N) or exerting toxic effects (Figure 2K), except in the spleen. Interestingly, aminotransferase and creatinine levels were not affected by DHW‐221 treatment, whereas the alanine aminotransferase concentration was increased at high dose, indicating that DHW‐221 exerted only mild toxicity (Figure 2L). H&E staining also demonstrated that no abnormal pathological changes were present in the visceral organs (Figure 2M). Moreover, DHW‐221 treatment could ameliorate the burden of orthotopic carcinoma to nude mice (Figure 2O, P). These findings indicated that DHW‐221 exhibited significant anti‐NSCLC activity with low toxicity and few side effects both in vitro and in vivo. Meanwhile, we investigated the pharmacokinetic of DHW‐221 in mice, and the parameters are provided in Table S3. With an oral bioavailability of up to 67.5%, the overall PK parameters of DHW‐221 appear promising and encourage its further evaluation for antitumor study.

**FIGURE 2:**
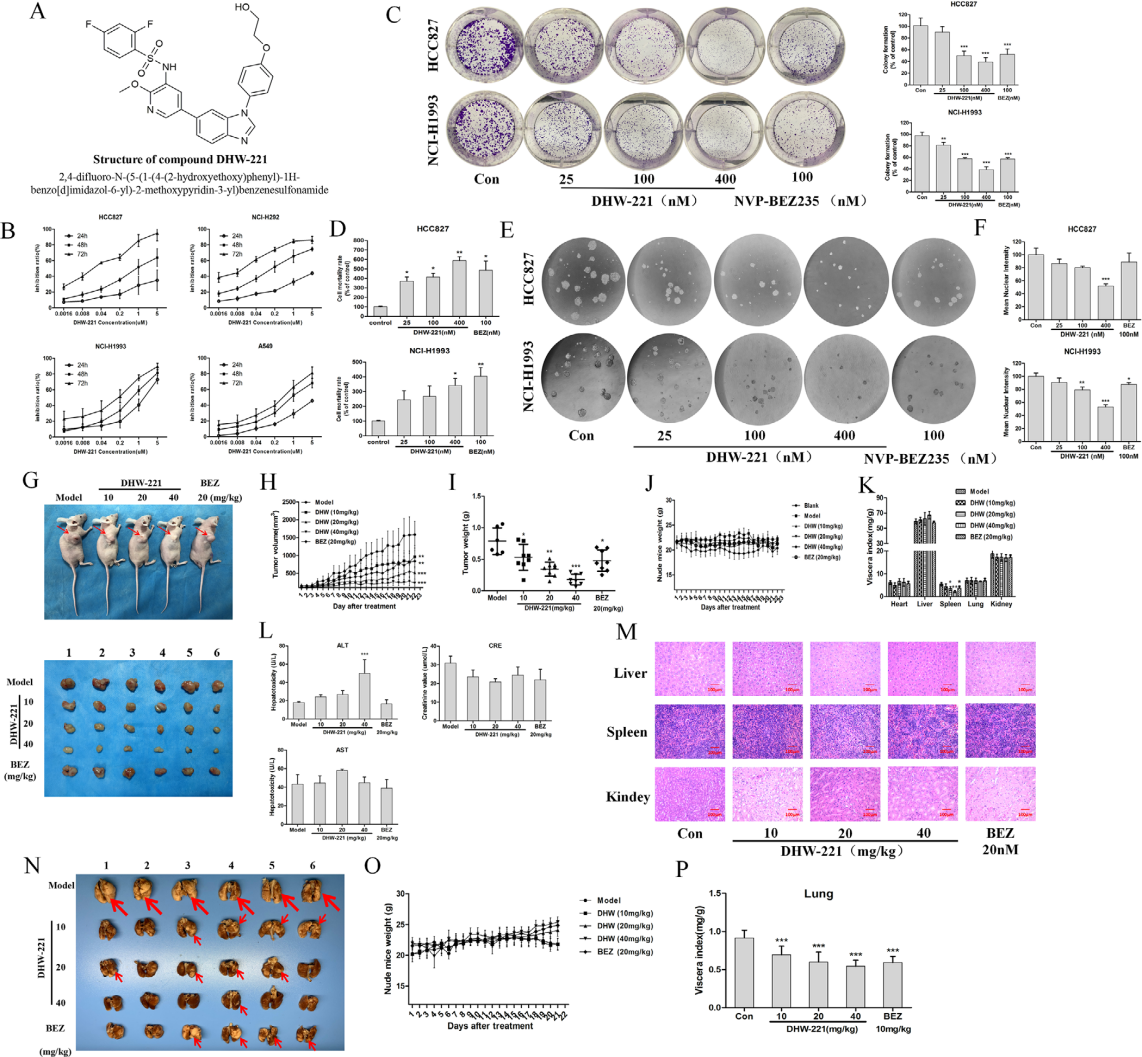
DHW‐221 inhibits the viability of NSCLC cells both in vitro and in vivo. (A) The structure of compound DHW‐221. (B) The viability of HCC827, NCI‐H292, NCI‐H1993, and A549 cells treated with various concentrations of DHW‐221 at 24, 48, and 72 h was determined by sulforhodamine B (SRB) assay. (C–E) The growth‐inhibitory effect of DHW‐221 on HCC827 and NCI‐H1993 cells was measured using a colony formation assay (C), a lactate dehydrogenase (LDH) assay (D), and a tumor sphere formation assay (E). The right panel in (C) shows the quantitative results for the colony formation assay. (F) OPP‐labeled protein content was detected by luciferase plate. (G) Tumor‐burdened nude mice and resected tumor samples from subcutaneous xenografts after 21 days (*n *= 6). (H) Subcutaneous tumor volume was measured daily with a caliper. (I) Mean tumor weight was measured to subcutaneous xenografts at the end of the experiment. (J) Subcutaneous xenografts nude mice were weighed daily on an electronic scale. (K) The index of each main organ of subcutaneous xenografts nude mice. (L) Liver and kidney function indexes subcutaneous xenografts nude mice were evaluated to predict drug toxicity. (M) Hematoxylin and eosin (H&E) staining of the liver, spleen, and kidney from subcutaneous xenografts nude mice; scale bar = 100 µm. (N) Photographs of NSCLC orthotopic tumor samples, *n* = 6. (O) Orthotopic xenografts nude mice were weighed daily on an electronic scale. (P) The index of lung organ was measured from orthotopic xenografts nude mice. Mean ± SD; *p < 0.05, **p < 0.01, ***p < 0.001 versus the control (model). One‐way analysis of variance followed by Tukey's post hoc multiple‐comparisons test

The PI3K/AKT/mTOR pathway is strongly linked to the regulation of cell cycle progression and apoptosis during cell proliferation.^6^, ^7^ In Figure 3A, NSCLC cells were arrested at G0/G1 phase by DHW‐221 concentration‐dependently. Meanwhile, the expression of endogenous cyclins in G0/G1 phase was detected by western blot, confirming that DHW‐221 regulated the cell cycle (Figure 3B). Apoptosis is crucial for anticancer drugs to kill cancer cells.^7^ First, Hoechst 33342 and Annexin V/PI staining verified that DHW‐221 concentration‐dependently induced apoptosis (Figure 3C, D). Moreover, transmission electron microscopic analysis of cellular ultrastructure also suggested that cells treated with DHW‐221 had typical apoptotic characteristics (Figure 3E). Additionally, JC‐1 staining showed that the mitochondrial membrane potential was concentration‐dependently decreased, further confirming that DHW‐221 induced cell apoptosis via the mitochondrial pathway (Figure 3F). Finally, the proapoptotic effects of DHW‐221 were confirmed by western blot analysis of apoptosis‐related protein expression both in vitro and in vivo (Figure 3G–I).

**FIGURE 3:**
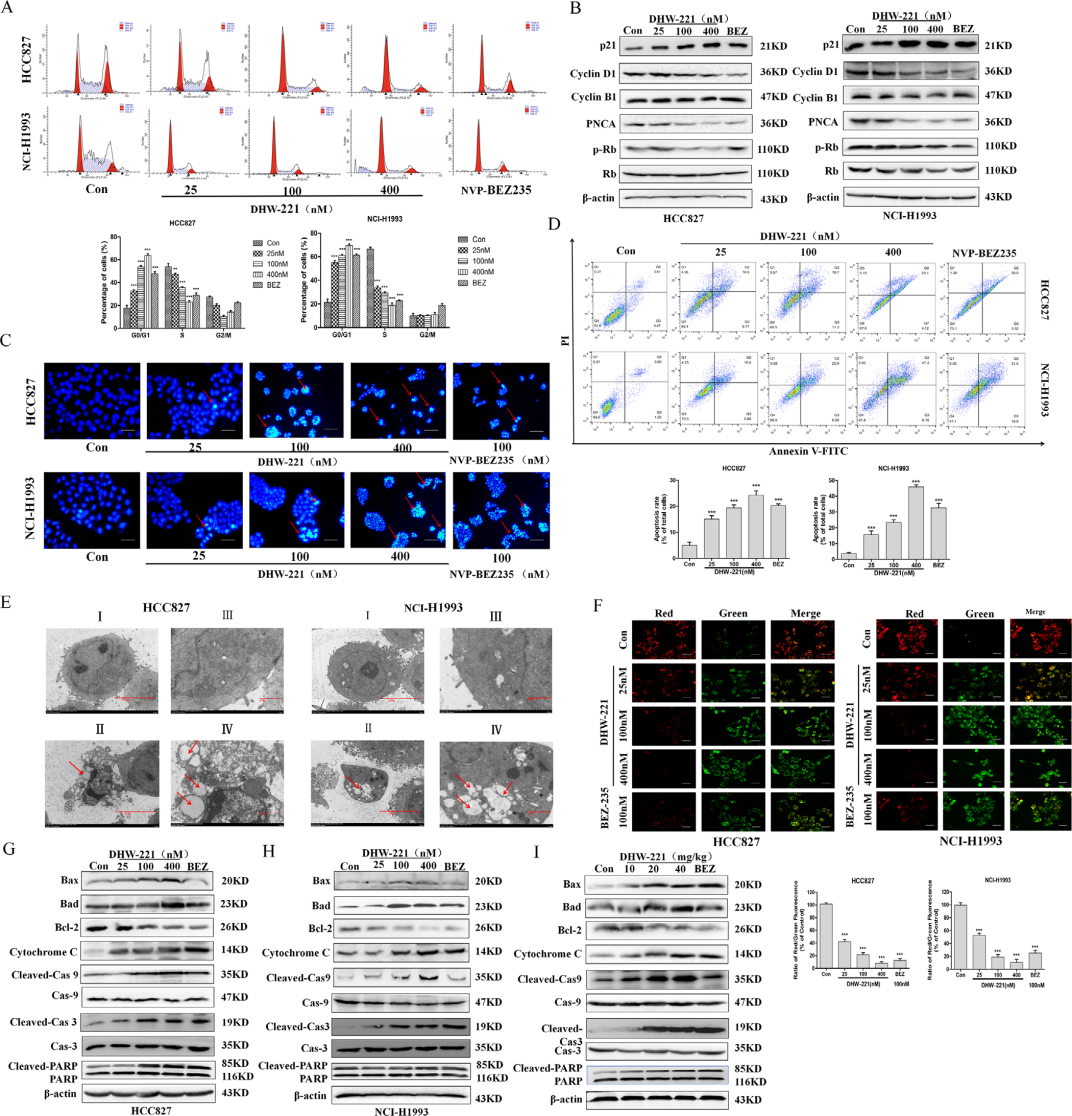
DHW‐221 induces cell cycle arrest and cell apoptosis in NSCLC both in vitro and in vivo. (A) DHW‐221 and NVP‐BEZ235 were detected by flow cytometry for 48 h. The raw histogram is shown at the top and the quantification of specific cell cycle stages at the bottom. (B) Western blot analysis of the expression levels of cell cycle‐related proteins in HCC827 and NCI‐H1993 cells after 48 h of treatment with the specified concentrations of DHW‐221 and NVP‐BEZ235. (C) Hoechst 33342 staining results. Apoptotic cells are bright blue; scale bar, 50 µm. (D) Analysis of the apoptotic effects of DHW‐221 and NVP‐BEZ235 by Annexin V/propidium iodide (PI) staining. The histograms show the percentage of apoptotic HCC827 and NCI‐H1993 cells treated with DHW‐221 or NVP‐BEZ235 for 48 h (lower panel). (E) Transmission electron microscopic analysis of the morphological changes occurring in cells after 48 h of treatment (I and II: scale bar = 5 µm [left]; III and IV: scale bar = 2 µm [right]). (F) The results of JC‐1 staining. Green fluorescence indicates decreased mitochondrial membrane potential, red fluorescence indicates normal mitochondrial membrane potential; scale bar, 100 µm. The histogram shows the average ratio of red/green fluorescence analyzed by Image J (bottom). (G–I) The expression levels of apoptosis‐related proteins in NSCLC cells (G, H) and tumor tissues (I) were detected by western blot. Mean ± SD; *p < 0.05, **p < 0.01, ***p < 0.001 versus the control. One‐way analysis of variance followed by Tukey's post hoc multiple‐comparisons test

Activated PI3K/AKT/mTOR signaling participates in tumor metastasis, invasion,^8^ and angiogenesis.^9^ In Figure 4A and B, DHW‐221 exerted a greater capacity in curtailing the migration and invasion of NSCLC cells. Meanwhile, western blot confirmed that DHW‐221 downregulated the expression of invasion‐related factor MMP‐2 (Figure 4C). Notably, migration and differentiation (tube formation) of endothelial cells are necessary for early angiogenesis.^10^ DHW‐221 markedly inhibited the metastatic (Figure 4D, E) and invasive (Figure 4E) potential of HUVEC and concentration‐ and time‐dependently suppressed the tube formation (Figure 4F). Additionally, DHW‐221 concentration‐dependently suppressed microvessel sprouting in the rat aortic ring ex vivo (Figure 4G) and reduced chorioallantoic membrane neovascularization in vivo (Figure 4H). Matrigel plug assay also suggested that DHW‐221 could significantly inhibit angiogenesis in vivo (Figure 4I). VEGFA and HIF‐1α both play key roles in tumor angiogenesis; here, DHW‐221 could concentration‐dependently inhibited the expression of these two proteins (Figure 4J, K). Together, these data indicate that DHW‐221 can reduce tumor angiogenesis by inhibiting the PI3K/HIF‐1α/VEGF signaling pathway.

**FIGURE 4:**
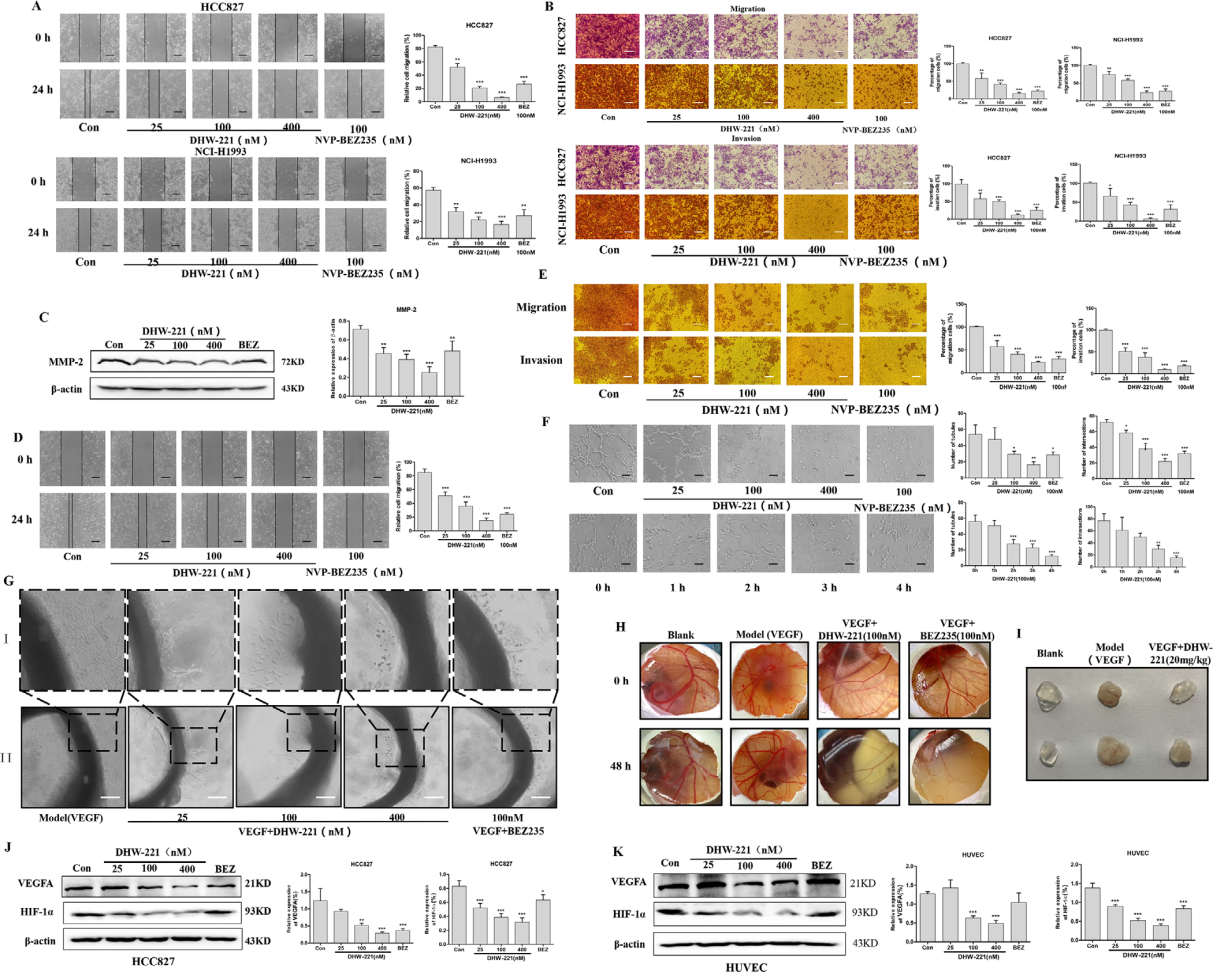
DHW‐221 inhibits cell migration, invasion, and angiogenesis in vitro, ex vivo, and in vivo. (A, D) The migratory ability of NSCLC cells (A) and HUVECs (D) was tested by wound healing assay; scale bar, 50 µm. A histogram of the migration rates is shown on the right. (B, E) The invasive ability of NSCLC cells (B) and HUVECs (E) was assessed by Transwell assay; scale bar, 50 µm. Right panel: Histogram showing the average number of cells calculated in five random fields on each filter under each condition. (C) The expression level of the MMP‐2 protein was detected by western blot; a bar graph of the quantitative results is shown on the right. (F) DHW‐221 inhibited tube formation in HUVECs. Left panel: representative images; scale bar, 100 µm; right panel: histogram showing the average number of cells in five random fields on each filter under each condition. (G) DHW‐221 inhibited microvascular sprouting in rat aortic rings; scale bar, 100 µm. (H) Chicken eggs were treated with vehicle, VEGF (50 ng/ml), or VEGF+DHW‐221 or NVP‐BEZ235. (I) Matrigel plugs containing vehicle, VEGF (50 ng/ml), or VEGF+DHW‐221 were implanted into mice, and photographs were taken after 7 days to determine the extent of angiogenesis. Representative images from separate experiments are shown. (J, K) Western blot results of VEGFA and HIF‐1α expression in HCC827 and HUVEC cells treated with DHW‐221. A bar graph of the quantitative results is shown on the right. Mean ± SD; *p < 0.05, **p < 0.01, ***p < 0.001 versus the control. One‐way analysis of variance followed by Tukey's post hoc multiple‐comparisons test

In conclusion, DHW‐221 was identified as being a dual PI3K/mTOR inhibitor, and displayed excellent anti‐NSCLC activity, including the inhibition of cell proliferation, migration, invasion, and angiogenesis, by blocking the PI3K/AKT/mTOR signaling pathway both in vitro and in vivo. Additionally, DHW‐221 was found to promote cell cycle arrest and mediate apoptosis through the mitochondrial pathway. Combined, these findings indicate that DHW‐221 has potential as a novel anticancer drug targeting PI3K/mTOR in NSCLC to inhibit tumor initiation and progression.

## CONFLICT OF INTEREST

The authors declare no conflict of interest.

## ETHICS APPROVAL AND CONSENT TO PARTICIPATE

All animal experiments were conducted in accordance with the guidelines of the internationally recognized guidelines for animal studies: reporting in vivo experiments. This study was approved by the Ethics Committee for Animal Experiments of Shenyang Pharmaceutical University.

## AUTHOR CONTRIBUTIONS

Prof. Q.C. Z. and Dr. H.W. D. for supervision and funding acquisition; X.C. Q. for conceptualization, methodology, validation, formal analysis, writing‐original draft, writing‐review and editing, and project administration; M.Y. L. and Y.T. W. for investigation and resources; others for editing and proofread. All authors read and approved the final manuscript.

## DATA AND MATERIALS AVAILABILITY

All data needed to evaluate the conclusions in the paper are present in the paper and/or the Supplementary Materials. Additional data related to this paper may be requested from the authors. Used research material is available by request from the corresponding author or from commercial sources when applicable.

## Supporting information

Supporting information
