# Supplementary material for: Dual blocking of PI3K and mTOR signaling by DHW‐221, a novel benzimidazole derivative, exerts antitumor activity in human non‐small cell lung cancer
Source: Clin Transl Med. 2021 Sep 26;11(9):e514. doi: 10.1002/ctm2.514 (PMC8473641; doi:10.1002/ctm2.514)
Supplement: Supplementary file 1 — Supporting information [file CTM2-11-e514-s001.docx]

**Supplementary material**


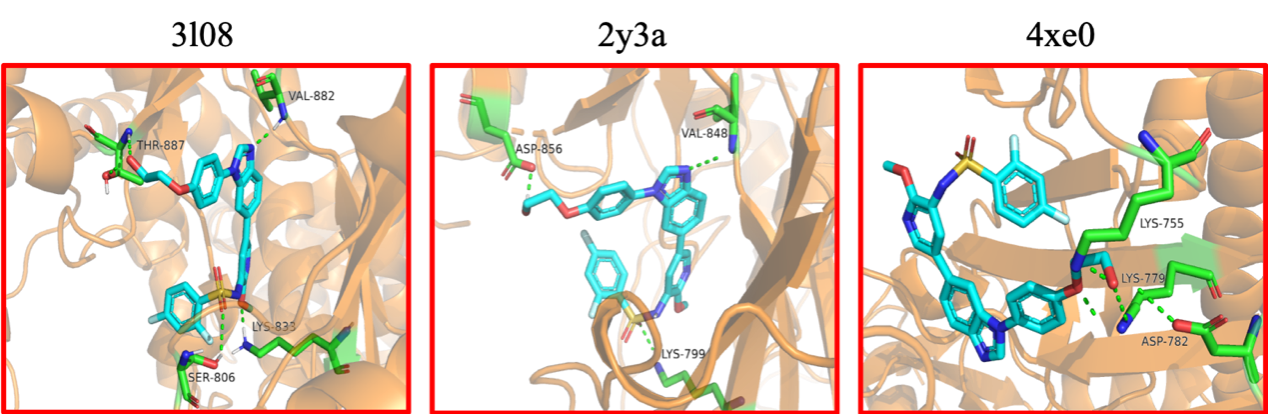


**Fig.S1** PI3K and mTOR are direct targets of DHW-221. Predicted binding modes of DHW221(blue stick) with PI3Kβ (PDB code: 2Y3A), PI3Kγ (PDB code:3L08) and PI3δ (PDB code:4XE0). Hydrogen bonds are shown as green dashed lines. Key residues interacting with the DHW221 are highlighted.

**Table S1** IC_50_ of DHW-221 against mTOR kinase *in vitro*.

| Compound | mTOR(nM) |
| --- | --- |
| DHW-221 | 3.9 |
| PI-103^a^ | 21 |

^a^ PI-103 served as a positive control.

**Table S2** IC_50_ of DHW-221 against human NSCLC cell lines (mean±SD, n=3)

| Cell line |  | IC_50_ (uM) |  |
| --- | --- | --- | --- |
|  | 24h | 48h | 72h |
| HCC827 | 8.331±1.451 | 0.970±0.127 | 0.032±0.018 |
| NCI-H292 | 7.323±2.418 | 0.253±0.149 | 0.026±0.003 |
| NCI-H1993 | 3.233±1.293 | 0.762±0.083 | 0.117±0.054 |
| A549 | 7.137±1.244 | 0.893±0.288 | 0.372±0.086 |

**Table S3** Pharmacokinetics Parameters of DHW-221^a^

| Parameters | T1/2(h) | Tmax(h) | Cmax(ng/mL) | AUCall(h·ng/mL) | MRTinf_obs(h) | F(%) |
| --- | --- | --- | --- | --- | --- | --- |
| Value (pob) | 7.1±0.9 | 3.0±1.7 | 11761.7±3400.3 | 176495.5±51062.0 | 11.4±0.3 | 67.5% |
| Value (ivb) | 3.7±0.2 | 0.083 | 67395.0±8545.0 | 261501.9±75698.9 | 4.7±0.3 |  |

^a^ Values are the average of three runs. Vehicle: DMSO:ethanol:saline=3:100:897. ^b^ Dose:10 mg/kg. T_1/2,_ half-life; C_max_, maximum concentration; T_max_, time of maximum concentration; AUC_all_, area under the plasma concentration time curve.

**Result S1 DHW-221 effectively blocked the notch signaling pathway**

The PI3K signaling pathway is interrelated with Notch. However, the regulatory relationship between Notch and PI3K/AKT/mTOR pathways is still unclear. Next, we investigated the effects of DHW-221 on Notch and its downstream critical target Hes-1. As shown in Figure S2，DHW-221 concentration-dependently inhibited the expression of Notch1(FL), Notch(NTM) and downstream protein Hes-1 in both HCC827 and NCI-H1993 cell lines, but had no effect on Notch2 and Notch3, indicating that DHW-221 could inhibit the expression of total Notch1 protein and activated fragment NICD，thereby inhibiting Notch pathway.


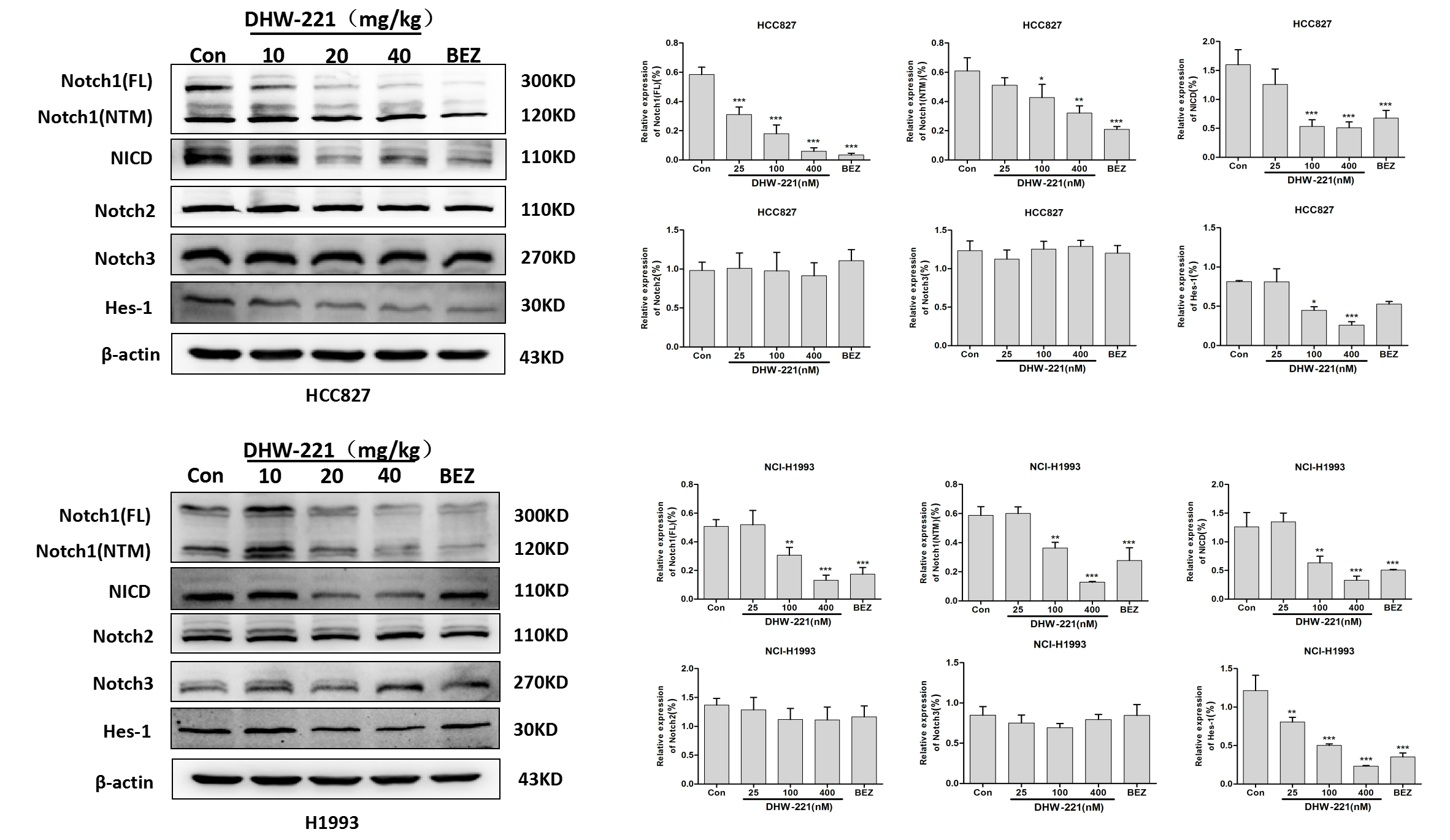


**Figure S2.** DHW-221 blocks the notch pathway. HCC827 and NCI-H1993 cells were treated with DHW-221 or NVP-BEZ235 for 48 h following which western blotting was performed on the cell lysates. Mean ± SD; ***p <* 0.01, ****p <* 0.001 *vs*. the control. One-way analysis of variance followed by Tukey’s post hoc multiple-comparisons test.


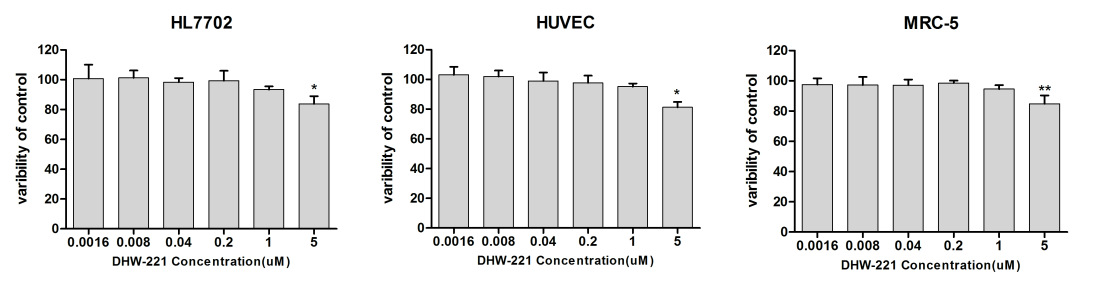


**Figure S3.** The effect of DHW-221 on normal cell lines. SRB assay was used to determine the cytotoxicity of DHW-221 toward normal cell lines (HL7702, HUVEC, and MRC-5) at 72 h. Mean ± SD; ***p <* 0.01, ****p <* 0.001 *vs*. the control. One-way analysis of variance followed by Tukey’s post hoc multiple-comparisons test.
